# Targeted Quantification of Detergent-Insoluble RNA-Binding Proteins in Human Brain Reveals Stage and Disease Specific Co-aggregation in Alzheimer’s Disease

**DOI:** 10.3389/fnmol.2021.623659

**Published:** 2021-03-18

**Authors:** Qi Guo, Eric B. Dammer, Maotian Zhou, Sean R. Kundinger, Marla Gearing, James J. Lah, Allan I. Levey, Joshua M. Shulman, Nicholas T. Seyfried

**Affiliations:** ^1^Department of Biochemistry, School of Medicine, Emory University, Atlanta, GA, United States; ^2^Goizueta Alzheimer’s Disease Research Center, School of Medicine, Emory University, Atlanta, GA, United States; ^3^Department of Pathology and Laboratory Medicine, School of Medicine, Emory University, Atlanta, GA, United States; ^4^Department of Neurology, School of Medicine, Emory University, Atlanta, GA, United States; ^5^Departments of Neurology, Neuroscience and Molecular & Human Genetics, Baylor College of Medicine, Houston, TX, United States; ^6^Jan and Dan Duncan Neurological Research Institute, Texas Children’s Hospital, Houston, TX, United States

**Keywords:** amyloid beta-protein, tau, RNA-binding proteins, Parkinson’s disease, Alzheimer’s disease, parallel reaction monitoring, mass spectrometry, human brains

## Abstract

Core spliceosome and related RNA-binding proteins aggregate in Alzheimer’s disease (AD) brain even in early asymptomatic stages (AsymAD) of disease. To assess the specificity of RNA-binding protein aggregation in AD, we developed a targeted mass spectrometry approach to quantify broad classes of RNA-binding proteins with other pathological proteins including tau and amyloid beta (Aβ) in detergent insoluble fractions from control, AsymAD, AD and Parkinson’s disease (PD) brain. Relative levels of specific insoluble RNA-binding proteins across different disease groups correlated with accumulation of Aβ and tau aggregates. RNA-binding proteins, including splicing factors with homology to the basic-acidic dipeptide repeats of U1-70K, preferentially aggregated in AsymAD and AD. In contrast, PD brain aggregates were relatively depleted of many RNA-binding proteins compared to AsymAD and AD groups. Correlation network analyses resolved 29 distinct modules of co-aggregating proteins including modules linked to spliceosome assembly, nuclear speckles and RNA splicing. Modules related to spliceosome assembly and nuclear speckles showed stage-specific enrichment of insoluble RBPs from AsymAD and AD brains, whereas the RNA splicing module was reduced specifically in PD. Collectively, this work identifies classes of RNA-binding proteins that distinctly co-aggregate in detergent-insoluble fractions across the specific neurodegenerative diseases we examined.

## Introduction

Protein aggregation in the brain is one of the major pathologic hallmarks of neurodegenerative diseases (Taylor et al., 2002; Ross and Poirier, 2004). These aggregates cause both functional deficits and toxicity that is thought to lead to neurodegeneration. Detergent-insoluble β-amyloid (Aβ) in senile plaques and intracellular accumulation of neurofibrillary tangles (NFTs) composed of tau are the core pathologies of Alzheimer’s disease (AD) (Masters et al., 1985; Grundke-Iqbal et al., 1986). Recent evidence indicates that detergent-insoluble RNA-binding proteins (RBPs) also accumulate and aggregate in AD (Bai et al., 2013; Diner et al., 2014; Hales et al., 2014, 2016; Bishof et al., 2018). For example, we previously discovered that a core component of the spliceosome complex, U1 small ribonucleoprotein 70 kDa (U1-70K), becomes insoluble in both symptomatic and early pre-symptomatic stages of AD where individuals had high brain amyloid pathology, yet were cognitively normal at death (Bai et al., 2013; Hales et al., 2016). The insolubility of additional U1 small nuclear ribonucleoprotein (snRNP) proteins, including U1-70K, was also observed in individuals with early-onset familial forms of AD caused by mutations in amyloid precursor protein (APP). Collectively this suggests that U1 snRNP aggregation in both familial and late-onset sporadic AD follows the loss of APP homeostasis and Aβ deposition (Hales et al., 2014). However, U1-70K does not co-aggregate with extracellular Aβ plaques in AD brains, yet in neurons mis-localizes from the nucleus to the cytoplasm were it co-aggregates with NFTs (Diner et al., 2014; Hales et al., 2016; Bishof et al., 2018; Hsieh et al., 2019). These co-aggregates between related U1 snRNP proteins and tau appear to be specific to AD as they are not observed in other tauopathies (Bai et al., 2013; Diner et al., 2014; Hales et al., 2016; Bishof et al., 2018). This supports a hypothesis that the cytoplasmic aggregation of U1-70K and functionally related RBPs provide a biological link between Aβ deposition and tau aggregation in AD.

Emerging evidence indicates that a diverse number of RBPs in addition to U1 snRNP proteins may also play an important role in mediating pathways of tau aggregation and subsequent neurodegeneration (Diner et al., 2014; Wolozin and Apicco, 2015; Bishof et al., 2018; Johnson et al., 2018; Maziuk et al., 2018; Raj et al., 2018; Hsieh et al., 2019). Notably, heterogeneous RBPs are found to mislocalize to the cytoplasm and co-aggregate with tau, which include the stress induced RBPs TIA1 (Vanderweyde et al., 2012; Vanderweyde et al., 2016) and G3BP that are reported to increasingly aggregate as disease severity progresses even in the absence of classic markers of tau pathology. Several of these RBPs that aggregate in neurodegenerative disease contain low complexity (LC) domains, including U1-70K, TDP-43, and FUS (Kwong et al., 2007; Neumann et al., 2009; Cohen et al., 2012). Our group and others have shown that U1-70K can aggregate independent from tau through self-assembly driven by multivalent interactions between structurally disordered basic-acidic dipeptide (BAD) LC domains (Diner et al., 2014; Bishof et al., 2018; Xue et al., 2019). However, whether other aggregation events occur systematically across an entire RBP class, or among specific RBPs expressed in human brain, is not clear.

Amyloidogenic and fibril-like aggregates, like Aβ and tau in AD, respectively, are highly resistant to biochemical or thermal denaturation and solubilization (Ramirez-Alvarado et al., 2000). It is therefore a challenge to purify and analyze aggregates through conventional biochemical methods (Ross and Poirier, 2004; Woltjer et al., 2005; Gozal et al., 2009; Seyfried et al., 2012; Diner et al., 2014; Nizhnikov et al., 2014; Hales et al., 2016). Ionic detergents such as N-lauryl-sarcosine (sarkosyl) can be used to enrich misfolded protein aggregates (Woltjer et al., 2005; Gozal et al., 2009; Diner et al., 2014, 2017; Hales et al., 2016). Despite solubilizing the majority of natively folded proteins in brain, sarkosyl is unable to solubilize aggregated proteins, which are pelleted out of solution following ultracentrifugation. The sarkosyl-insoluble pellet can be solubilized by a strong chaotropic agent like urea for subsequent proteomic analysis, revealing significant enrichment of β-amyloid or tau as well as RBPs such as U1-70K in AD brain (Seyfried et al., 2012; Bai et al., 2013; Diner et al., 2017). Emerging evidence demonstrates that specific subclasses of RBPs are insoluble in different neurodegenerative disease homogenates (Hasegawa et al., 2002; Miake et al., 2002; Neumann et al., 2006; Hales et al., 2016; Bishof et al., 2018; Cherry et al., 2018). As previous studies link RBP aggregation to AD and other neurodegenerative diseases, we sought to perform a comprehensive quantification of relative RBP abundance in sarkosyl-insoluble protein fractions across brains with a varying degree of AD pathology and Parkinson’s disease (PD) as a disease control.

Mass spectrometry (MS) has emerged as an analytical tool to measure protein abundance in complex biological samples, providing excellent sensitivity, reproducibility and accuracy (Zhang et al., 2010; Gillette and Carr, 2013). In conventional discovery, or data-dependent acquisition (DDA) proteomic methods, the most abundant precursor peptide ions are selected for tandem MS analysis (MS/MS), which often biases measurement to the most abundant proteins in the sample. Thus, with highly complex proteomes, such as sarkosyl-insoluble brain extract, DDA approaches may result in some missing values across samples if the target of interest is not highly abundant. To circumvent these limitations, targeted data-independent acquisition (DIA) proteomic approaches such as parallel reaction monitoring (PRM) can be employed for sensitive and accurate quantification of peptides/proteins in brain tissue lysate (Peterson et al., 2012; Ronsein et al., 2015; Johnson et al., 2020). The PRM method can isolate and detect a select list of precursor ions simultaneously, mitigating abundance bias and minimizing missing peptide quantitation values.

Herein we describe a PRM method to monitor 870 peptides from 385 RBPs, plus microtubule-associated protein tau (MAPT), amyloid precursor protein (APP), Aβ, and internal standard peptides. We examined relative levels of these proteins in sarkosyl-insoluble fractions of 44 dorsolateral prefrontal cortex (DLPFC) tissues from control (CTL), AsymAD, AD, and PD cases. We confirmed the enrichment of Aβ, tau and U1-70K in the insoluble fractions of AsymAD and AD homogenates. In addition, several BAD RBPs showed enhanced insolubility in AD cases. We also discovered a variety of RBPs that were differentially reduced in the sarkosyl-insoluble fractions of AD or PD cases. A systems-level weighted protein correlation network analysis displayed biological coherence for enrichment of RBP complexes and module-wise differences across disease groups. Modules of spliceosomal snRNP and nuclear speckle proteins showed a stage-specific enrichment in the insoluble fraction of AsymAD and AD, whereas modules related to transcription and translation were depleted from the insoluble fraction of AD or PD brain extracts, suggesting failure of normal RBP complex formation or less aggregation of these RBP complexes specific to these diseases. Collectively, this work identifies numerous novel signatures of co-aggregating RBPs in neurodegenerative disease, particularly during discrete stages of AD, holding promise to define mechanistic events during neurodegeneration.

## Materials and Methods

### Materials

Primary antibodies used in this study include an in-house rabbit polyclonal antibody raised against a synthetic KLH-conjugated peptide corresponding to a C-terminal epitope of U1-70K (EM439) (Diner et al., 2014), a mouse monoclonal anti-tau phospho-threonine 231 antibody (clone MAB5450, EMD Millipore) and a rabbit polyclonal anti-TDP-43 antibody (10782-2-AP, ProteinTech Group). Secondary antibodies were conjugated to either Alexa Fluor 680 (Invitrogen) or IRDye800 (Rockland) fluorophores. Postmortem brain tissue from healthy control cases, pathologically confirmed AsymAD, AD, and PD cases were selected for comparison from the Emory Alzheimer’s Disease Research Center (ADRC) brain bank (*n* = 44). Tissue from Brodmann Area 9 of the dorsolateral prefrontal cortex (DLPFC) was isolated. Human postmortem tissue was acquired in accordance with Institutional Review Board (IRB) protocols at Emory University. The AsymAD and AD cases were defined as described before (Boros et al., 2017) with the neuropathological examination of plaque distribution performed according to CERAD (Mirra et al., 1991) and neurofibrillary tau tangle pathology staging conducted according to the Braak system (Braak and Braak, 1991). All AD cases met the NIA-Reagan criteria for the diagnosis of Alzheimer disease (high likelihood) (Hyman and Trojanowski, 1997). The PD post-mortem diagnoses were also made in accordance with established criteria and guidelines (McKeith et al., 1996, 2005; Brown et al., 1998; Braak et al., 2003). The DLB Neocortex score included is a qualitative binary measure based on whether neocortical Lewy body pathology was observed (1) or whether the Lewy body pathology did not meet the consensus criteria (0) previously described (Brown et al., 1998; McKeith et al., 2005). All of the PD cases indeed presented synuclein pathology at autopsy, and all individuals were clinically diagnosed with PD. Cases were matched as closely as possible for age at death, gender, and post-mortem interval. A summary of case characteristics is provided in Table 1. All case metadata is provided in Supporting Information (Supplementary Table 1).

**TABLE 1:** Characteristics of human subjects used in this study.

	Control	AsymAD	AD	PD
	**(*n* = 12)**	**(*n* = 8)**	**(*n* = 12)**	**(*n* = 12)**
**Age at Death,** mean years (SD)	76.0 (10.5)	82.5 (9.7)	76.4 (7.4)	75.1 (6.0)
**Disease Duration,** mean years (SD)			11.1 (4.0)	17.5 (9.6)
**Sex,** *n* (%)				
Male	6 (50.0)	5 (62.5)	8 (66.7)	9 (75.0)
Female	6 (50.0)	3 (37.5)	4 (33.3)	3 (25.0)
**Race,** *n* (%)				
White	9 (75.0)	8 (100.0)	12 (100.0)	12 (100.0)
Black	2 (16.7)	0 (0.0)	0 (0.0)	0 (0.0)
Hispanic	1 (8.3)	0 (0.0)	0 (0.0)	0 (0.0)
**PMI,** mean hrs (SD)	7.3 (3.9)	17.5 (9.6)	9.5 (8.2)	12.9 (7.6)
**Amyloid & Tau Burden**				
CERAD Score	0.2 (0.4)	2.8 (0.5)	3.0 (0.0)	0.2 (0.4)
Braak Score	1.6 (0.9)	3.0 (1.2)	5.9 (0.3)	2.2 (1.3)
ABC Score	0.5 (0.5)	1.8 (0.5)	3.0 (0.0)	0.4 (0.5)
**DLB Neocortex Score,** (SD)				0.5 (0.5)
**ApoE Status,** *n* (%)				
APOE2/2	0 (0.0)	0 (0.0)	0 (0.0)	0 (0.0)
APOE2/3	0 (0.0)	1 (12.5)	1 (8.3)	6 (50.0)
APOE2/4	0 (0.0)	1 (12.5)	0 (0.0)	0 (0.0)
APOE3/3	12 (100.0)	3 (37.5)	2 (16.7)	4 (33.3)
APOE3/4	0 (0.0)	2 (25.0)	6 (50.0)	2 (16.7)
APOE4/4	0 (0.0)	1 (12.5)	3 (25.0)	0 (0.0)

*AsymAD, Asymptomatic Alzheimer’s disease; AD, Alzheimer’s disease; PD, Parkinson’s disease; PMI. Post-Mortem Interval; CERAD, Consortium to Establish a Registry for Alzheimer’s disease; ABC: Amyloid/Braak/CERAD aggregate score (none = 0, low = 1, intermediate = 2, high = 3); SD, Standard Deviation.*

### Extraction of Detergent-Insoluble Fraction From Human Brain

Extraction was performed with a fractionation protocol using the ionic detergent N-lauryl-sarcosine (sarkosyl) essentially as described (Diner et al., 2017). Approximately 200 mg of brain tissue was used for each case and the whole extraction process was performed on ice (Supplementary Table 2). Brain tissues were homogenized with 0.9-2.0 mm stainless steel homogenization beads (Next Advance, SSB14B) in 1 ml of low salt buffer (50 mM HEPES pH 7.0, 250 mM Sucrose and 1mM EDTA pH7.4 in H_2_O) and 1× HALT protease/phosphatase inhibitor cocktail (Thermo Fisher Scientific) with a Bullet Blender^®^ (Next Advance) for 2 × 5 min, generating a total homogenate (TH) lysate. A total of 800 μl of TH was aliquoted to a separate tube and mixed with 100 μl of 5M NaCl and 100 μl of 10% Sarkosyl (N-lauroylsarcosine), giving final concentrations of 0.5M NaCl and 1.0% Sarkosyl. The total homogenate-sarkosyl mix (TH-S) was then sonicated with a microtip probe (Sonic Dismembrator, Thermo Fisher Scientific) at 30% amplitude 3 × 5 s to shear nucleic acids. Protein concentrations were determined using the bicinchoninic acid (BCA) method (Pierce). Five milligrams of TH-S was diluted with Sark Buffer (1% Sarkosyl and 0.5M NaCl in low salt buffer) to a total volume of 500 μl in 700 μl ultracentrifuge tubes. Tubes were balanced and centrifuged at 64,000 rpm for 30 min at 4°C to obtain an insoluble pellet. The supernatant was removed and saved, and the pellet was washed with 200 μl of Sark Buffer+1X HALT and centrifuged again. The resulting supernatant was discarded, and the pellet was washed with 500 μl PBS+1X HALT and centrifuged again. The final supernatant was discarded and 75 μl of 8M urea+1X HALT was added to solubilize the pellet, which was transferred into 1.5 ml Eppendorf tubes. Samples were then sonicated for 3 × 10 s on/off cycles at 20% amplitude to dissolve the pellet. The sonication was repeated every 30 min until the pellet was fully dissolved. The bicinchoninic acid assay (BCA) was performed to obtain protein concentration of the insoluble fraction. Insoluble fraction purity was then validated by western blot.

### Western Blotting

Western blotting was performed according to standard procedures as reported previously (Dammer et al., 2012; Seyfried et al., 2012; Diner et al., 2014). Samples were loaded at 25 μg per lane. Samples denatured in Laemmli sample buffer were resolved by SDS-PAGE before being semi-dry transferred to nitrocellulose membranes (Invitrogen) using the iBlot2 system (Life Technologies). Membranes were blocked with casein blocking buffer (Sigma B6429) for 30 min at room temperature and then probed with primary antibodies (see “Materials”) at a 1:1000 dilution overnight at 4°C. The next day, membranes were rinsed and incubated with secondary antibodies conjugated to Alexa Fluor 680 (Invitrogen) fluorophore at a 1:10000 dilution for 1 h at room temperature. Membranes were then rinsed and again incubated with secondary antibodies conjugated to a second 800 nm-emitting fluorophore at a 1:10000 dilution for 1 h at room temperature. Images were captured using an Odyssey Infrared Imaging System (Li-Cor Biosciences).

### Protein Digestion and Sample Preparation

A total of 30 μg of each sample was prepared in 100 μl of 8M urea buffer (10 mM-Tris, 100 mM NaH_2_PO_4,_ pH 8.5 with 1X HALT) for proteolytic digestion. Proteins were first reduced by DTT (final concentration of 1 mM) at room temperature for 30 min followed by alkylation with IAA (final concentration of 5 mM) in the dark at room temperature for 30 min. Proteins were then digested with Lys-C (enzyme ratio, 1:100) overnight at room temperature. The next day, samples were diluted 5 times to a final concentration < 2 M urea with 50 mM NH_4_HCO_3_ and digested with trypsin (enzyme ratio, 1:50) overnight at room temperature. Resulting peptides were desalted using an HLB column (10 mg, OASIS^®^) prior to liquid chromatography with tandem mass spectrometry (LC-MS/MS). A global pooled standard (GPS) sample was prepared for mass spectrometry analysis by pooling 10% of tryptic peptides from each case into a single tube. Then, 0.75 pmol/μl of 6 × 5 LC-MS/MS Peptide Reference Mix from Promega (6 peptides × 5 concentrations) was added into each of the 50 samples (44 case samples and 6 GPS technical replicates injected at different points throughout the sequence of 50 runs) before LC-MS/MS. The reference mix serves as an internal control to enable removal of any systematic variance. It is a mixture of 30 peptides: 6 sets of 5 isotopologues of the same peptide sequence, where the isotopologues of each peptide are present in a series of tenfold differences in molar abundance ranging from 0.0001X (lightest peptide) to 1X (heaviest peptide) (Promega, 2017).

### Parallel Reaction Monitoring (PRM)

To create the RBP inclusion list for PRM quantification, the global pooled standard (GPS) sample was first deeply profiled on an Orbitrap Fusion using the DDA method (Dai et al., 2018). Raw files were processed by Maxquant (Version 1.5.7.4), using a human whole proteome sequence FASTA file downloaded from UniProt (UP000005640, date: 02-16-2016) (UniProt Consortium, 2019). The search engine Andromeda was used. Protein methionine oxidation (+15.9949 Da), protein N-terminal acetylation (+42.0106 Da), phosphorylation (S/T/Y, +79.9663 Da) and lysine ubiquitination (K, +114.0429 Da) were searched for as variable modifications (up to five allowed per peptide); cysteine was assigned a fixed carbamidomethyl modification (+57.0215 Da). Only fully tryptic peptides were considered with up to two missed cleavages in the database search. A precursor mass tolerance of ±20 ppm was applied prior to mass accuracy calibration, and a ± 4.5 ppm tolerance was applied after internal MaxQuant calibration. Other search settings included a maximum peptide mass of 6,000 Da, a minimum peptide length of six residues, and 0.6 Da tolerance for ion trap HCD MS/MS scans. The false discovery rate (FDR) for peptide spectral matches, proteins, and site decoy fraction were all set to 1%. Following database searches, the identified peptides were used to build a PRM list of proteins that have gene ontologies related to RNA binding by filtering for gene symbols present in gene ontologies of “spliceosome,” “ribonucleoprotein,” “mRNA processing,” “RNA binding,” “small nuclear ribonucleoprotein and Sm protein,” in addition to the U1-70K interactome list generated by our group (Bishof et al., 2018). Peptides (and proteins) that were not suitable for protein quantification by unique peptide were removed. PRM analysis of peptides from the custom RBP list was then performed using a Q-Exactive Plus hybrid quadrupole-orbitrap mass spectrometer (Thermo Fisher Scientific), operated under Xcalibur software 3.0.63, equipped with a nano-electrospray ion source and coupled to a nano-Acquity system (Waters). Digested peptides were loaded and separated on a self-packed 1.9 μm C18 analytical column (New Objective, 70 cm × 75 μm inner diameter; 360 μm outer diameter). Elution was performed over a 160 min gradient at a rate of 240 nL/min with buffer B ranging from 1 to 99% (buffer A: 0.1% formic acid in water, buffer B: 0.1 % formic acid in acetonitrile). The Q-Exactive Plus MS was operated in PRM mode using an inclusion list, consisting of m/z values of targeted peptides (Supplementary Table 6). A normalized collision energy of 30% was employed for fragmentation. The following MS conditions were used: spray voltage, 2.0 kV; heated capillary temperature, 300°C; isolation windows, 1.6 m/z; resolution set to 17,500 at m/z 200; and an automatic gain control (AGC) target of 1 × 10^5^. The mass spectrometry proteomics data have been deposited to the ProteomeXchange Consortium via the PRIDE repository with the dataset identifier PXD022144 (Perez-Riverol et al., 2019).

### PRM Data Analysis

Before importing Thermo raw files into Skyline software (version 3.6), a spectral library was built. The library relied on global pooled standard (GPS) sample analyzed by an Orbitrap Fusion mass spectrometer described above with the same normalized collision energy as used for PRM analysis. For each peptide, three to six of the strongest product ions were selected in Skyline (MacLean et al., 2010). The Skyline software settings included: Precursor mass analyzer, centroided; MS1 mass accuracy of 20 ppm; Product mass analyzer, centroided; MS/MS mass accuracy of 20 ppm; Retention time filtering, including all matching scans. All product ion peak areas were calculated by Skyline, and endogenous peptide quantification was based on the normalized peak areas of at least 3 product ions. An example MS/MS spectrum of a tau peptide is included in Supplementary Figure 3.

### Data Normalization

Quantitative proteomic data including PRM can be affected by a variety of systematic biases, defined as non-biological signal, e.g., artifacts generated by the MS instrument from tuning parameters or chromatographic drift. Normalization of measured peptide or protein abundances must be robust to contend with this technical bias in LC-MS/MS data, including ionization efficiency drift, LC column and signal drift, and the interplay of these with static data acquisition timing (Karpievitch et al., 2012). Thus, to correct for technical bias in comparisons between samples, it is necessary to normalize abundance data. Systematic technical variance occurring across MS runs was removed by data normalization of the linear drift of heavy labeled internal standard peptide signals as described (Zhou et al., 2020). Originally, there was signal depreciation of standard peptides in the dataset across the series of 50 injections with a coefficient of variance (CV) of 27.08% (Supplementary Figure 4A). To control for signal attenuation, we applied a correction factor to each peptide peak area, thereby calculating normalized peptide peak areas (Supplementary Table 7). Normalized protein abundances were then calculated by summing corrected intensity peptide values (Supplementary Table 10). In the final normalized quantitative matrices of PRM abundances used for statistics, rare missing (zero) values (0.73% of all 17,160 protein abundance values) were imputed as noise at one half of the minimum non-zero abundance measured for the peptide or protein. Finally, these normalized protein abundances were log_2_-transformed and regressed for age, sex and PMI and used to compare protein insolubility across disease groups (Supplementary Table 11). As described above, an isotopically labeled heavy peptide reference mix from Promega was added as internal control for normalization. Since the amount of standard peptides in each sample were fixed at known quantities, the variation in signal intensity for these peptide standard curves generated across all samples was due to unwanted technical drift. We chose the reference peptide (with peptide molar abundance of 0.01x) to calibrate normalization of drift slope to 0 using four reference peptides (#2-5), which had linear signal changes across the sequence of MS runs and had the highest coefficient of determination values (R^2^) across all 50 samples. Correction factors for normalizing peptide/protein intensities were calculated using peak area. First, we averaged each of the four-reference peptide peak areas (intensities) Ī _P2_, Ī _P3_, Ī _P4_, and Ī _P5_ across all 50 injections, and then normalized each of the sample-reference-peptide peak areas Ā_*ij*_ (i = 2–5, j = 1–50) to an average of 1 by dividing Ā_*ij*_ by each corresponding peptide’s average intensity:

$$IntensityRatio\left(\text{IRij}\right)==\bar{\text{A}}\text{ij}/\bar{\text{I}}\text{Pi}$$

A scatter plot including normalized intensity ratio of peptides 2–5 across 50 runs, where the *x*-axis represented MS injections 1 to 50 and the *y*-axis represented the intensity ratios as calculated above, showed a linear relationship between injection number and intensity ratio with the following best-fit regression line (Supplementary Figure 4A):

y-0.0167x1.417,R2=0.769

This confirmed that peptide intensity across MS injections decreased due to signal depreciation. The average intensity ratio (Ī_*Rj*_) of each injection (*x* = 1 to 50) was calculated using the equation above. Then, intensity ratios of all run positions were adjusted so that a fit line would have a slope of zero (Supplementary Figure 4B), and we divided 1 by each Ī_*Rj*_ to obtain a multiplier correction factor, in turn applied to all original peptide intensities for each injection position j:

$$CorrectionFactor=1/\bar{\text{I}}\text{Rj}$$

After normalization, the CVs of four-reference peptides across 50 runs became 14.69% as compared to pre-normalization values (CVs = 27.08%, Supplementary Figure 4B).

### Protein Differential Abundance Analysis

Volcano plots were generated to visualize differential expression of RBPs in different case groups versus control. The *x*-axis, represented as log_2_ fold change, is the log_2_ ratio of average groupwise protein abundances in a pairwise comparison (e.g., AD/Control). Student *t*-test -log_10_(p) values represent statistical significance on the y-axes of volcano plots. Significantly altered proteins (*p*-Value < 0.05) are presented with *p*-Values and fold changes in Supplementary Table 12. For stacked bar plots, the fraction of co-expressed module members achieving differential abundance significance of *p* < 0.05 were counted (*y*-axis) for each of the indicated pairwise comparisons and coloring of the heat map scale was based on the range of minimum to maximum average log_2_ fold change of the significantly changed module member proteins. The limited sample size of our study limits power even without adjusting for multiple testing, so FDR adjustment of *p*-Values was not considered here.

### Correlation Network and Pathology Correlation Analysis

The R package Weighted Gene Correlation Network Analysis (WGCNA) v1.68 was used to cluster proteins by abundance into groups of co-expressed proteins using a dissimilarity metric for clustering distance based on 1 minus the topology overlap matrix (1-TOM), which is a calculation based on an adjacency matrix of correlations of all pairs of proteins in the abundance matrix supplied to WGCNA (Langfelder and Horvath, 2008). The power selected was the lowest power at which scale free topology R^2^ was approximately 0.80, or in the case of not reaching 0.80, the power at which a horizontal asymptote (plateau) was nearly approached before further increasing the power had no more or a negative effect on scale free topology R^2^. Other parameters were selected as previously optimized for protein abundance networks (Seyfried et al., 2017). Thus, for the signed network built on protein abundances obtained from PRM, parameters were input into the WGCNA::blockwiseModules() function as follows: Beta (power) 23, signed network type, biweight midcorrelation (bicor), mergeCutHeight = 0.07, pamStage TRUE, pamRespectsDendro TRUE, reassignThreshold *p* < 0.05, minKMEtoStay = 0.30, deepSplit = 4, minModuleSize = 5, TOMdenom = “mean,” and maxBlockSize greater than the total number of proteins. To correlate RBP abundances to pathological aggregated insoluble Aβ and tau abundances, bicor rho values were calculated and reported.

### Gene Ontology Enrichment and Co-clustering Analysis

GO-Elite v1.2.5 used a Fisher exact test *p* < 0.05 for determining significance of hypergeometric overlap and a minimum of 3 gene symbols per GO Term. The background insoluble proteome consisted of all 4313 gene symbols represented in a MaxQuant (Andromeda) search performed as described above on data-dependent acquisition data for the insoluble global pooled standard protein sample obtained on both Orbitrap Fusion and Q-Exactive mass spectrometers. Z-score equivalent of significant two-tailed Fisher exact test *p*-Values, i.e., *Z* > 1.96, was the threshold for over-representation of ontologies. Z-score bar graphs were plotted in R. The full table of Fisher exact test significance values and transformed as Z scores for cellular component ontologies enriched in any of the 29 WGCNA modules was also calculated by GO-Elite v1.2.5 using the same total insoluble proteome background. This Z-score table was co-clustered using the R NMF package a heatmap function, implementing Manhattan distance function, with a Ward metric.

## Results and Discussion

### Enrichment and Targeted Mass Spectrometry Quantification of Detergent-Insoluble RNA-Binding Proteins Across Neurodegenerative Diseases

**FIGURE 1:**
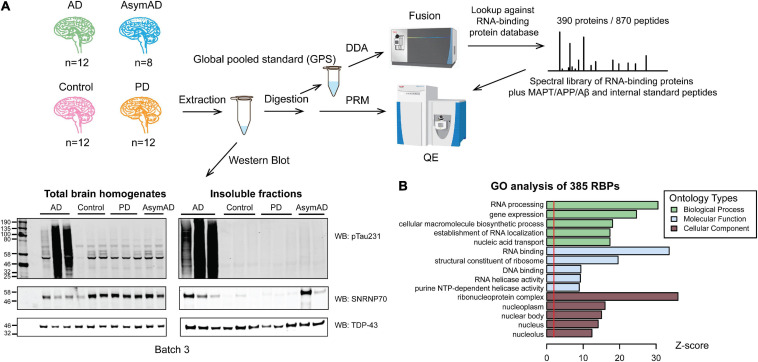
Workflow of the targeted PRM method for quantification of RNA-binding proteins in detergent-insoluble fractions of human brains. **(A)** Workflow beginning with dorsolateral prefrontal cortex brain samples from control group (*n* = 12), Alzheimer’s disease (AD, *n* = 12), Asymptomatic AD (AsymAD, *n* = 8), and Parkinson’s disease (PD, *n* = 12) from which insoluble fractions were extracted and digested for subsequent quantification. Western blotting total homogenates and insoluble fractions of each group (*n* = 3, each) for TPD-43 (loading control), phosphorylated tau (pThr231-tau) and U1-70K indicates successful insoluble fraction extraction. A global pooled standard (GPS) was deeply profiled to identify the total insoluble proteome using an Orbitrap Fusion mass spectrometer. A custom targeted RNA-binding protein (RBP) peptide list and corresponding spectra library was designed for PRM, including 870 peptides from 385 RBPs plus tau, Aβ, APP and internal standard peptides. Finally, PRM was run on Q-Exactive mass spectrometer for all 44 samples using 6 GPS samples to normalize between batches. **(B)** GO analysis of detected 385 RBPs linked proteins to “RNA processing” and “RNA binding” biological processes and molecular functions, respectively.

We enriched protein aggregates by sarkosyl detergent fractionation of dorsolateral prefrontal cortex (DLPFC) tissue in 44 cases of control, AsymAD, AD and PD cases (Figure 1A). Cases were matched as closely as possible for age at death, gender, and post-mortem interval (PMI) (Table 1 and Supplementary Table 1). PD cases served as a disease control, which do not show consistent evidence of U1 snRNP aggregation (Bai et al., 2013). Wet tissue amounts and insoluble protein yields were summarized to show no significant difference in the relative protein amounts of insoluble fractions between case groups (Supplementary Table 2). The aggregate enrichment was validated by western blot of pT231-tau, a marker of neurofibrillary tangles, and U1-70K (Figure 1A and Supplementary Figure 1A), counterposed with TDP-43 labeling as a loading control. Insolubility of tau and U1-70K are hallmarks of AD post-mortem brain (Bai et al., 2013; Hales et al., 2014, 2016). A large signal increase of insoluble pT231-tau was observed only in AD cases (Supplementary Figure 1B), while U1-70K was enriched in the insoluble fraction of both AsymAD and AD (Supplementary Figure 1C), with little to no enrichment of either hallmark observed in PD. These data are consistent with previous findings that showed insolubility of U1 snRNP components including U1-70K in early stages of preclinical AD, preceding that of tau (Hales et al., 2016).

After successfully enriching for insoluble proteins across different neurodegenerative disease cases, we comprehensively characterized the total insoluble proteome to build a target list of observable aggregating RNA-binding proteins (RBPs) across all disease groups. To generate the target list, a global pooled standard (GPS) consisting of an equivalent amount of tryptic peptides from all samples was analyzed by LC-MS/MS in data-dependent acquisition (DDA) mode. Of all the identified insoluble proteins, we prioritized 385 proteins involved in RNA biology and RNA binding (RBPs, see definition in “Methods” Section “Parallel Reaction Monitoring (PRM)”). As expected, gene ontology (GO) analysis of the custom 385 RBP list compared against the insoluble proteome used as background (Figure 1B) revealed predominant GO terms associated with the “RNA processing” biological process and the “RNA binding” molecular function. Furthermore, we observed an enrichment of “ribonucleoprotein complex,” and “nucleus”-related cellular component GO terms, indicating that we captured a broad range of RBPs across divergent compartments of the cell. Using Skyline software (MacLean et al., 2010), we developed a targeted mass spectrometry-based PRM method to analyze 870 peptides from 385 RBPs, plus tau, APP and Aβ, and internal control spike-in peptides essentially as described (Zhou et al., 2019, 2020). Three hundred twenty four out of 390 proteins were common to previously published stochastic data dependent acquisition (untargeted) single-shot LC-MS/MS data (Supplementary Figure 2A; Johnson et al., 2020). About 97% of these 324 proteins have 0-5% missing PRM values, compared to 54% of proteins in the untargeted data (Supplementary Figure 2B). These analyses highlight the value of the targeted PRM method for quantifying RBPs with significantly less missingness than in an untargeted approach.

The PRM method achieved relative quantification of each target RBP peptide across all 44 case-samples, by which we could calculate abundance changes of each target RBP across different disease groups. A representative spectrum of a peptide from tau (IGSTENLK, residues 260-267) was selected to show how peptides were quantified by PRM (Supplementary Figure 3). Several fragment ions were detected from each peptide in the MS/MS spectra. The top five y-ions of the peptide were selected for PRM analysis (Supplementary Figure 3A). Peptides were quantified by integrating chromatographic areas of multiple fragment ions at the MS/MS level to determine relative abundance across the case-samples (Supplementary Figure 3B). At least three fragment ions were used to quantify each peptide using Skyline. The IGSTENLK tau peptide, mapping to the first repeat (R1) of the microtubule binding region (MTBR), was enriched specifically in AD group samples. This peptide is within the pronase-resistant region of tau and is a likely constituent of the protofilament core of AD tau (Fitzpatrick et al., 2017). Also, the IGSTENLK tau peptide harbors one of the KXGS motifs that regulate tau aggregation in a post-translational modification-dependent manner (Cook et al., 2014). Therefore, insoluble fraction abundance changes measured by PRM at the peptide level reflect insolubility or aggregation changes unique to heterogeneous diseases. We have also compared the signal intensities of western blot densitometry measurements to PRM quantification of pT231-tau (cor = 0.92, *p* = 1.1e–18, Student’s p for Pearson rho; Supplementary Figure 3C) and U1-70K protein (cor = 0.81, *p* = 2.7e–11; Student’s p for Pearson rho, Supplementary Figure 3D), observing a significant degree of correlation between the independent measurement procedures. This highlights the utility of PRM analysis for the interrogation of disease-specific attributes of the insoluble RBP proteome.

### Region and Modification-Specific Peptides From APP and Tau Exhibit Increased Insolubility in AD

**FIGURE 2:**
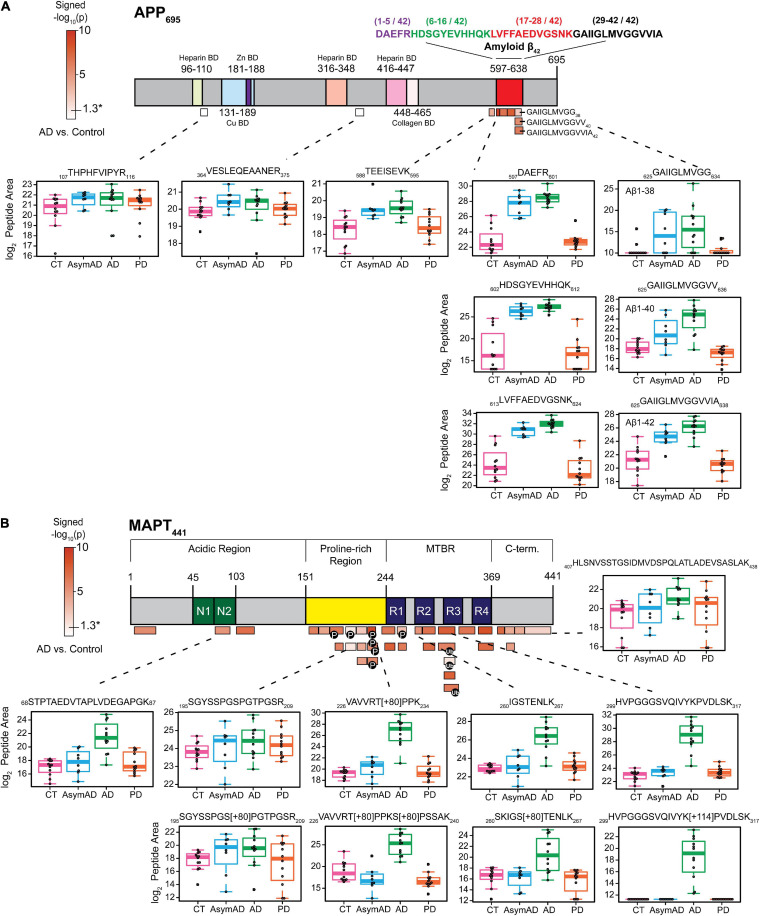
Specific peptides from Aβ and tau exhibit disease-associated abundance changes in the detergent-insoluble fractions. **(A)** The schematic of APP_695_ where 9 peptides were identified and quantified across all 44 samples. The color scale represented -log10(*t*-test *p*) signed values, calculated on log_2_-transformed data for AD vs. control case group comparison only. Subdomains identified from the UniProt database were marked with different colors. Peptides with significantly altered insoluble fraction abundances (*p*-Value < 0.05) are all mapped to the Aβ region. Box plots show the significant enrichment of Aβ peptides in AsymAD and AD while peptides mapping elsewhere in APP are unaltered across disease groups. **(B)** Peptides (*n* = 35) were mapped to the MAPT_441_ (2N4R) and colored according to their corresponding *p*-Value as described above. Most identified peptides showed significant differences between AD and control group (*p*-Value < 0.05).

Amyloid beta plaques and tau neurofibrillary tangles are core pathological hallmarks of the AD brain (Glenner and Wong, 1984; Iqbal et al., 1984). To further confirm enrichment of insoluble protein species in our sarkosyl-insoluble, aggregate-enrichment method, we asked whether we could measure changes in this aggregate-enriched fraction at the peptide level. We created a schematic of the neuronal APP_695_ isoform, where all targeted peptides by PRM were aligned to their corresponding region in the protein. Mapped peptides were colored according to -log_10_ *t*-test *p*-Values of insoluble fraction abundance differences between the AD group and controls (Figure 2A and Supplementary Table 7). Individual boxplots of examined peptides across the entire APP_695_ sequence were generated to directly show differences in peptide abundance from the same protein across all disease groups. Notably, *y*-Values used in the boxplots were log_2_ transformed peptide area and those peptide areas with a zero value were imputed in order to perform statistical analysis (Supplementary Table 7). The imputation and log_2_ transformation compressed differences between groups contributing to more conservative *p*-Values (Supplementary Tables 7, 8). Still, peptides of Aβ and tau protein showed significant enrichment in AD cases. We observed that among the nine peptides mapping to APP, only those mapping to the Aβ region (residues 597-638) were highly enriched in AD (Figure 2A), consistent with previous findings (Seyfried et al., 2017), whereas peptides from the N-terminus of APP were unchanged in AD versus control cases. Enrichment of the Aβ-derived peptides in the insoluble fraction of AD brain is due to the AD-specific appearance of amyloid plaque aggregates (Seyfried et al., 2017) as a consequence of proteolytic cleavage by β- and γ-secretases, respectively (Vassar et al., 2009; Chen et al., 2017). Boxplots of all peptides mapping to APP further illustrate the differential regulation of peptide-level insolubility across different case groups. Whereas Aβ-derived insoluble peptides exhibited strong, stage-specific increased enrichment in AsymAD and AD, they were unchanged in PD, demonstrating the ability of our method to distinguish AD-specific amyloid aggregation.

Similarly, the 35 peptides mapping to tau (MAPT) (Figure 2B) show varying degrees of increased abundance in AD insoluble fractions compared to control. Peptides from the MTBR region were highly enriched in AD versus control cases. The MTBR region forms the highly insoluble and stable core of neurofibrillary tangles (Fitzpatrick et al., 2017), which are required for seeding tau aggregation. Phosphorylation and ubiquitination are PTMs that modulate signaling pathways and pathophysiological mechanisms in AD. Unsurprisingly, these classes of PTMs decorate the MTBR region of tau and are enriched in AD (Abreha et al., 2018; Ping et al., 2018, 2020; Arakhamia et al., 2020). Although highly enriched in AD, tau peptides were largely unchanged in AsymAD, demonstrating the late aggregation of tau protein during progression of AD pathology. This is in agreement with Braak staging and extensive molecular imaging and proteomic protein quantification studies in AD brain (Braak and Braak, 1991; Gozal et al., 2009; Bateman et al., 2012; Villemagne et al., 2013; Hales et al., 2016), and further highlights the reliability of our PRM method to detect and quantify changes in the insoluble proteome specific to AD.

### Differential Aggregation of RNA-Binding Proteins Across Neurodegenerative Diseases

**FIGURE 3:**
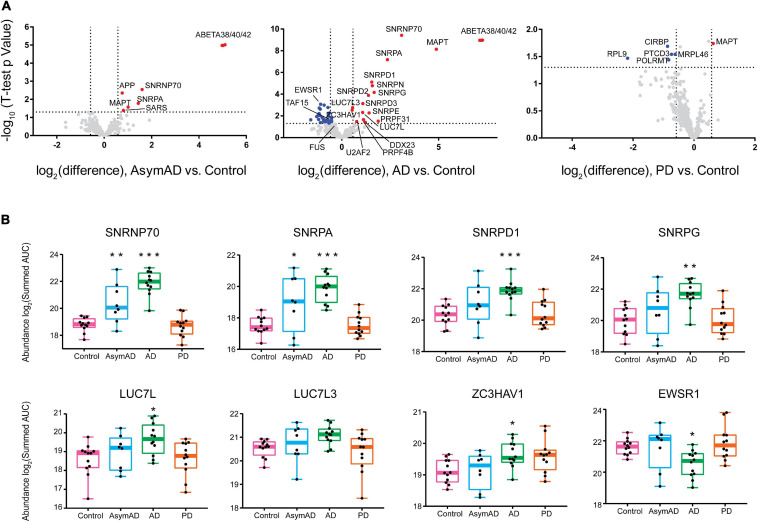
RNA-binding proteins have different abundance patterns in the detergent-insoluble fractions across neurodegenerative diseases. **(A)** Differential abundance of RBPs across different groups was shown by volcano plot containing 385 RBPs, plus tau, APP and total β-amyloid. Fold-change, displayed on the *x*-axis, was the log_2_ protein abundance ratios for pairwise comparisons (AsymAD/Control, AD/Control, PD/Control). The t-statistic (−log10(*p*-Value)) was calculated for all proteins in each pairwise group and displayed on the *y*-axis. Insoluble fraction enriched proteins were highlighted in red (| Fold Change| > 1.5, *p*-Value < 0.05) while proteins with decreased insolubility were highlighted in blue, and gray dots represented proteins that remained unchanged. Several RBPs, including splicing factors, showed increased insolubility in AsymAD and AD while RBPs in PD were largely decreased in the insoluble fraction. **(B)** ANOVA boxplots of disease-signature RBPs (^∗^*p* < 0.05, **p < 0.01, ^∗∗∗^*p* < 0.001). SNRNP70 and SNRPA showed stage-specific increase in detergent-insoluble fraction of AsymAD and AD; SNRPD1, SNRPG, LUC7L, and ZC3HAV1 only showed significant increase in AD insoluble fractions; FET family member EWSR1 exhibited a decreased abundance in AD insoluble fractions.

Although several neurodegenerative diseases are distinguished by RBP aggregation (Bai et al., 2013; Ramaswami et al., 2013; Diner et al., 2014; Hales et al., 2014; Wolozin and Apicco, 2015; Maziuk et al., 2017), different diseases contain aggregates of distinct classes or families of RBPs. To examine whether there were disease-specific RBP aggregation events in the brain proteomes of our cohort, we generated volcano plots of three pairwise comparisons of neurodegenerative disease groups versus controls (Figure 3A). Insoluble RBPs with a significant alteration in abundance within a disease group were considered as those beyond a 50 percent fold change (±1.5) with a *p*-Value < 0.05. A complete list of differentially insoluble RBPs is listed in Supplementary Table 11 (*t*-test for control vs. disease) and Supplementary Table 12 (ANOVA for all pairwise comparison). A few representative RBPs with significant changes in Figure 3A were shown in Figure 3B. Notably, U1 snRNP proteins SNRPA and U1-70K (SNRNP70) exhibited significant enrichment within the insoluble fraction of AsymAD brain, along with Aβ. This finding confirms our previous work that links U1 snRNP pathology to AD (Bai et al., 2013; Diner et al., 2014; Hales et al., 2014; Bishof et al., 2018), which coincides with Aβ plaque deposition. These events are likely to occur earlier than insoluble tau accumulation, which is primarily enriched in the insoluble fraction of symptomatic AD samples. Interestingly, tau is elevated in PD group as well. Other snRNP component RBPs belonging to the Sm ring of the spliceosome, including SNRPN, SNRPD1, SNRPD2, SNRPD3, SNRPE and SNRPG exhibit significant and robust insolubility increase in AD brain. In addition to core snRNP component RBPs, the splicing-associated RBPs LUC7L and LUC7L3, as well as U2AF2, DDX23 and PRPF4B, each with BAD repeats homologous to those of U1-70K, are enriched among insoluble proteins of AD-confirmed cases (Bishof et al., 2018; Kundinger et al., 2020). This suggests that proteins with BAD domains shift towards insolubility by way of some unknown mechanism in AD. In contrast, a noteworthy group of heterogeneous nuclear ribonucleoproteins (hnRNPs) called the FET (FUS/EWSR1/TAF15) family is more soluble in AD brain. In addition, a variety of RNA-binding proteins involved in protein translation or transcription were observed to be depleted preferentially from the insoluble fraction of PD cases, including pentatricopeptide repeat domain-containing protein 3 (PTCD3), 60S ribosomal protein L9 (RPL9), mitochondrial ribosomal protein L46 (MRPL46), cold-inducible mRNA binding protein (CIRBP) and DNA-directed RNA polymerase (POLRMT). This suggests a shift of RBPs involved in translation and transcription away from the insoluble fraction, specific to PD—though it is also commensurate with a possible decrease of these proteins in PD regardless of insoluble fraction enrichment. As we only measured the insoluble fraction, we were limited in the power to interpret an enrichment or depletion in the insoluble fraction of RBPs in the disease groups. Possible reasons for the significant depletion of an RBP from the insoluble fraction include increased degradation, preferential chaperone-mediated folding mechanisms or reduced expression of that protein (Bukau et al., 2006; Cohen et al., 2006; Balch et al., 2008). Significant enrichment of specific RBPs may also be dependent on the magnitude of their abundance in the total proteome. However, as we previously showed there were no obvious spliceosome changes via core snRNPs like U1-70K or U1A observed in the total proteome, and rather, bulk changes are driven by cell types instead of individual proteins (Higginbotham et al., 2020). We hypothesize the enrichment of core snRNP components in insoluble fractions is not driven by their abundance in the total proteome and may be considered as reflective of the insolubility shift of these proteins in specific disease groups. Nonetheless, the disease-specific increases and decreases in insoluble fraction enrichment of different groups of RBPs suggests that different protein-mediated RNA metabolism events are dysregulated in different neurodegenerative diseases (Liu et al., 2017). Our findings reveal several novel signatures of co-aggregating RBPs suggesting that RBPs are differentially dysregulated across different neurodegenerative diseases, leading to diverse subsequent pathology, molecular pathophysiology, and symptoms.

### Correlation Network Analysis Groups Detergent-Insoluble RNA-Binding Proteins Into Modules With Shared Biological Function

**FIGURE 4:**
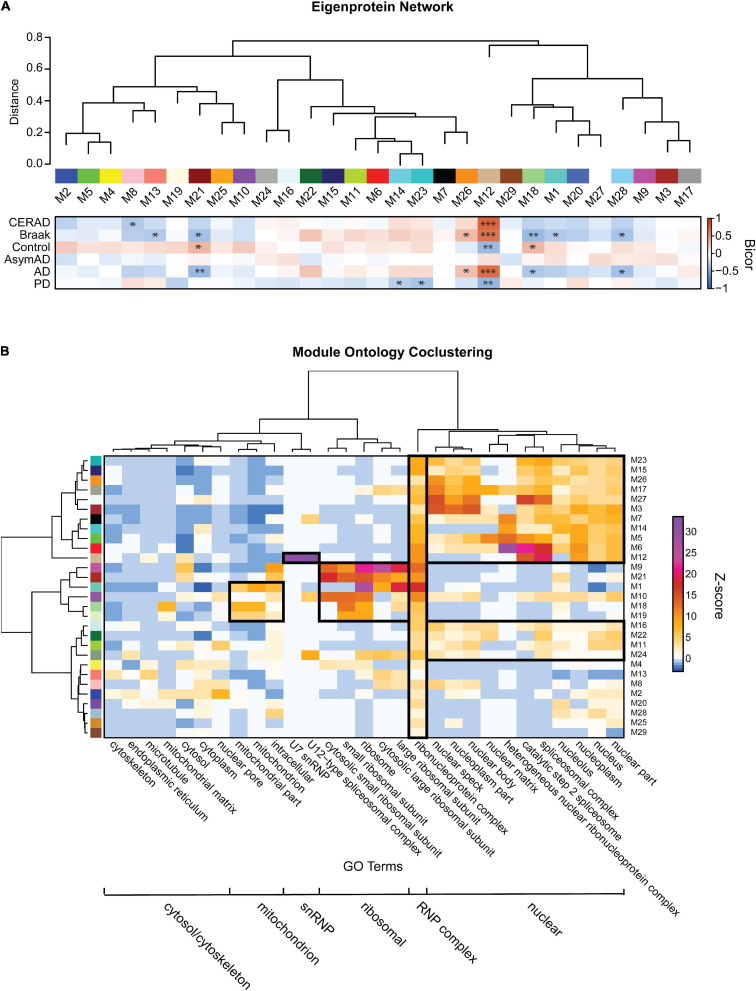
Detergent-insoluble protein co-abundance network and ontology clusters. **(A)** 29 RBP modules were obtained by WGCNA network analysis. Modules were visualized in relatedness order, with the heatmap shown representing robust bicor correlation of each module to CERAD/Braak scores or each disease diagnoses. Bicor correlations are illustrated as a directional heatmap with a color scale and asterisks to display significance (^∗^*p* < 0.05, ^∗∗^*p* < 0.01, and ^∗∗∗^*p* < 0.001). Correlations between each module was shown by eigenprotein network indicated on the top. **(B)** For each module, a Z-score for Gene Ontology (GO) terms was calculated which reflects the over-representation of the components of that module to each GO term. Six “clusters” of RBP modules were grouped according to their over-representation of GO-terms involved in mitochondrion, snRNP, ribosomal, RNP, nuclear and cytoplasmic complexes.

To further study the biology underlying co-aggregation between different RBPs, we performed a systems biology analysis using WeiGhted Co-expression Network Analysis (WGCNA) to cluster RBP abundances within sarkosyl-insoluble fractions across our multiple neurodegenerative disease brain cohort. The network reduced the data into 29 modules based on correlation (rank-ordered by size, M1-M29) that were each assigned a different color (Figure 4A). These modules are made up of proteins that highly correlated to each other which typically reflects their similarity in the biology or structure. An eigenprotein network was generated to show correlations between modules. An eigenprotein is defined as the first principal component of a given module and serves as a representative, weighted expression profile for the module (Seyfried et al., 2017). Individual RBP module membership is listed in Supplementary Table 14. Correlation of each module to post-mortem CERAD/Braak scores or disease states were illustrated as a heatmap, with significance visualized by a color scale and asterisks (*^∗^p* < 0.05, ^∗∗^*p* < 0.01, ^∗∗∗^*p* < 0.001). The M12 module, which contains tau and Aβ as well as all snRNP components measured, was the most highly correlated with CERAD scores (standard assessment of AD plaque pathology) and Braak staging (of tangle pathology extent). The M12 module insolubility profile across the cohort was also positively correlated with AD diagnosis (*p* < 0.001). The eigenproteins that drive the insolubility co-abundance of M12 are all snRNP components (SNRPN, U1-70K, U1A, SRNPD1/3, SNRPG). These proteins are highly correlated with each other and with amyloid deposition even in early, preclinical stages of AD, consistent with previous observations (Hales et al., 2016). Another module, M26, containing the BAD proteins LUC7L, LUC7L3, DDX23 and RBM39, was also positively correlated with AD diagnosis and increased Braak staging. In agreement with the depletion of insoluble RBPs observed in PD (Figure 3A), there were several modules (M12, M14 and M23) with insolubility profiles that were anti-correlated to PD status. Therefore, we were able to observe biologically coherent groups of RBPs that correlated with disease status and relevant traits.

To reveal functionally distinct groups of modules, and thereby simplify the granularity of our network, we grouped RBP modules by their strength of association to different GO terms, using co-clustering analysis (Figure 4B). This illustrated the discrete enrichment of RBP modules to six different “macro” clusters. These included nuclear complexes, ribosomal units, RNP complexes, snRNPs, mitochondrion and cytoplasmic complexes. Because our hypothesis-driven MS approach targeted RBPs, every module grouped into the RNP complex cluster. A separate nuclear cluster consisted of a large group of RBP modules, as the nucleus is a hub for many membrane-less RBP organelles (Boeynaems et al., 2018). The M12 module, consisting almost entirely of snRNP component RBPs plus Aβ and tau, grouped into a separate snRNP cluster. Interestingly, the M12 module grouped with the nuclear cluster but adjacent to the mitochondrial and ribosomal clusters. This may recapitulate known subcellular localization and varied protein-protein interaction behaviors of snRNP proteins (Fischer et al., 1993, 1997; Romac et al., 1994; Liu et al., 1997; Bishof et al., 2018). The ribosome and mitochondrial clusters consisted of some of the same modules (M1, M10, M18, and M19), consistent with mitochondria utilizing independent translation machinery. Interestingly, one of these modules (M18) is significantly depleted from the insoluble fraction of AD brain, compared to control brain. In addition, the ribosome- and mitochondria-associated M19 module was depleted from the insoluble fraction in PD samples compared to control. These changes of translation machinery in the disease groups may indicate dysfunction of translation and links a recent finding of crosstalk between translation inhibition and aggresomal formation, involving nuclear capped-RNA-binding proteins like NCBP1 (Park et al., 2018). Notably, NCBP1, RAE1, XPOT, and EIF5A are involved in the passage of mature mRNAs from the nucleus to cytoplasm through nuclear pores, and all four of these proteins are members within M2, a module uniquely enriched for the gene ontology “nuclear pore.” Trait correlations for M2 (Figure 4A) trend such that only control cases in the cohort have enhanced levels of insoluble M2 proteins, suggesting that the nuclear pore basket may be compromised in AD and PD. Taken together, these findings suggest that ribosome complexes and translation machinery may be less stable as large insoluble complexes in neurodegenerative diseases, perhaps downstream of defective mature mRNA export. Overall, by using bioinformatic and systems biology approaches, we were able to group RBPs and modules together to infer shifts in biological function specific to the different neurodegenerative diseases represented in our cohort.

### Distinct Classes of Aggregated RNA-Binding Proteins Show Disease Specificity

**FIGURE 5:**
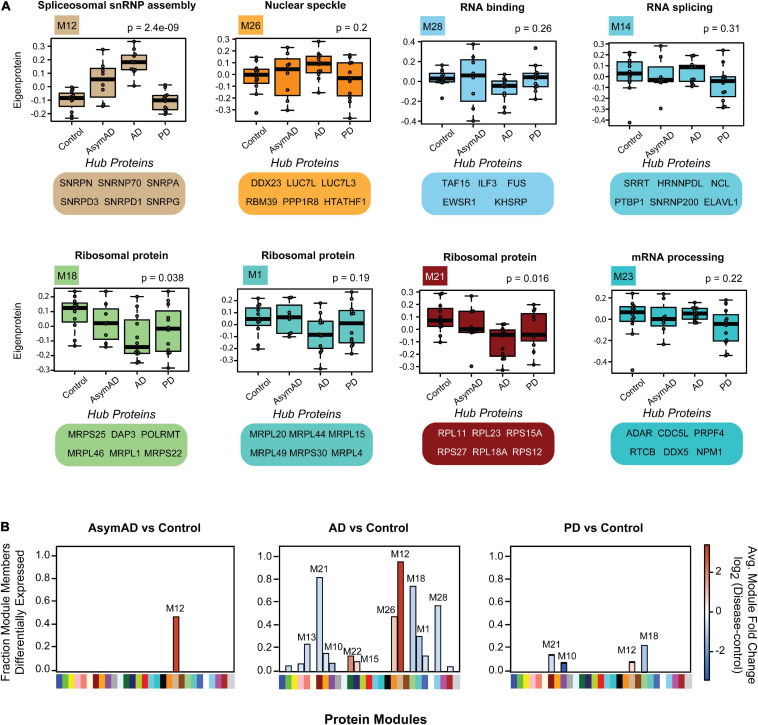
Module-wise insolubility changes according to disease status. **(A)** A highlight of modules that were significantly correlated with disease groups. Top GO terms were highlighted for each module. All of 29 individual modules overrepresent gene ontologies. For example, M12 shows a group of proteins that belong to “spliceosome snRNP assembly;” M26 includes “nuclear speckle” proteins. The top six proteins which drive the module insolubility, called eigenproteins, are listed below each box plot. M12 and M26 showed stage-specific enrichment in detergent-insoluble fraction of AsymAD and AD. Modules M18, M21 and M28 exhibited decreased insolubility in AD. Modules M14 and M23 experience decreased insolubility in PD only. The central horizontal bar in each box is the 50th percentile (median), and hinges of the box extents represent the interquartile range of the two middle quartiles of data within a group. The farthest data points, up to 1.5 times the interquartile range away from box hinges, define the extent of whiskers (error bars). Significance was measured using one-way non-parametric ANOVA, Kruskal-Wallis *p*-Values. **(B)** Module eigenprotein levels for each pairwise comparison between disease groups and controls are shown. Direction of solubility change is colored according to log_2_ fold change colored scale. The percentage of module members that were differentially abundant in the insoluble fraction in disease compared with control is plotted on the *y*-axis.

To illustrate module-specific insolubility changes across disease groups, we generated box plots of insoluble proteome eigenproteins (Figure 5A). M12, with hubs involved in spliceosomal snRNP assembly, including U1-70K, SNRPA and other snRNPs-as well as Aβ and tau-was increasingly insoluble-enriched in control, AsymAD, and AD cases. M26, another key module the insolubility of which correlates with AD diagnosis, is enriched with GO term “nuclear speckle” proteins. Importantly, M26 includes the BAD proteins LUC7L, LUC7L3, DDX23 and RBM39 (Bishof et al., 2018; Kundinger et al., 2020). Given the positive correlation with AD diagnosis and CERAD and Braak scores of these RBP modules, we sought to rank RBPs by their correlation to the Aβ and tau insoluble protein profiles we measured across the 44 case samples of our cohort. To assess this, we calculated biweight midcorrelation (bicor) coefficients for all 385 RBPs as pairwise protein comparisons to Aβ _(1–42)_ and tau insoluble fraction abundances across all 44 individual cases (Supplementary Figures 5A,B and Supplementary Table 15). Strikingly, the entire M12 module consisted of the highest correlated RBPs to both Aβ and tau insolubility. In addition, the next best ranking members included BAD proteins LUC7L, LUC7L3, and DDX23 of M26. This suggests that spliceosome associate RBPs may be a bridge between Aβ and tau aggregation pathologies in AD.

Among the RBPs most anti-correlated to insoluble tau abundance include the mitochondrial proteins MRPL46 and PTCD3, which are significantly depleted from the insoluble fractions of PD. Notably, tau protein facilitates an interaction between MRPL46 and stress-related RBP TIA1, and this interaction is eliminated when tau expression is lost (Vanderweyde et al., 2016). TIA1 and other stress granule RBPs including G3BP were not enriched in the sarkosyl-insoluble fractions in this work, consistent with previous studies (Vanderweyde et al., 2012; Wolozin, 2012; Hales et al., 2016). Instead, TIA1 preferentially stabilizes soluble oligomeric tau in stress granules, whereas TIA1 reduction promotes accumulation of fibrillar tau (Vanderweyde et al., 2016; Apicco et al., 2018). This highlights the potential of sarkosyl-soluble RBPs to regulate the aggregation of tau in addition to other RBPs. Some modules comprised of proteins that are anti-correlated to Aβ and tau show decreased insolubility in both AD and PD, such as M18, M21 and M28. The M28 module contains FET family members FUS/EWSR1/TAF15, and the other two are comprised of ribosomal subunit proteins. Over 50% of the members of M18, M21 and M28 are differentially soluble in AD (Figure 5B). As memory formation requires constant and activity-dependent adaptive protein biosynthesis, ribosomal protein solubility changes may indicate neuronal dysfunction and cognitive decline, consistent with a decrease in overall translation within neuronal cells (Meier et al., 2016; Koren et al., 2019). Two modules (M14 and M23) related to mRNA processing and RNA splicing, respectively, are specifically depleted from the insoluble fractions of PD brain, which is consistent with a shift in solubility dynamics specific to PD. Overall, we have identified modules representing solubility changes specific to neurodegenerative diseases that can be leveraged to focus future investigations and hypotheses regarding of RBP-associated pathogenic and mechanisms tied to aggregation propensity of these proteins.

## Conclusion

In this work, we developed a targeted MS method to quantify aggregated RBPs in neurodegenerative disease brain tissue. In total we analyzed ∼900 peptides mapping to 385 RBPs in addition to tau, Aβ, APP and internal standard peptides, and examined their relative abundance across detergent-insoluble fractions of 44 individual dorsolateral prefrontal cortex tissue samples of patients with pathologically confirmed diagnoses of control, AsymAD, AD or PD. Our findings recapitulate the known disease specific aggregation of tau and β-amyloid in AD progression. Additionally, we have found numerous novel co-aggregating RBPs across these disease groups and have discussed their potential pathological mechanisms involved in either initiation or progression of pathophysiology within these diseases. The insoluble RBPs with differential abundance across different groups might represent potential novel protein-RNA complexes that are targets for therapeutic development. Moreover, further studies targeting this panel of insoluble RBPs across other neurodegenerative disease, including tauopathies, may reveal additional links between tau pathology and co-aggregation RBPs. Finally, we reported a strong correlation between U1 snRNP proteins and AD pathology which supports a hypothesis that U1 snRNP aggregation provides a bridge between amyloid deposition with tau aggregation. The targeted approaches described in this study collectively hold promise in defining mechanistic events during distinct neurodegenerative states perturbing RBP homeostasis in the human brain, to be deciphered in future studies.

## Data Availability Statement

The datasets presented in this study can be found in online repositories. The names of the repository/repositories and accession number(s) can be found below: http://www.proteomexchange.org/, PXD022144.

## Author Contributions

QG and MZ carried out the experiments. QG, MZ, SK, and ED performed the data analyses. ED did the computational analyses. QG drafted the original manuscript and figures. QG, SK, ED, and NS wrote and edited the manuscript. NS supervised the project. QG, ED, MZ, SK, MG, JL, AL, JS, and NS reviewed and edited the manuscript. AL, JS, and NS carried out the funding acquisition. MG collected the resources. All authors contributed to the article and approved the submitted version.

## Conflict of Interest

The authors declare that the research was conducted in the absence of any commercial or financial relationships that could be construed as a potential conflict of interest.

## Supplementary Material

Supplementary Figure 1 Western blot and PRM measurements for pT231-tau and U1-70K are highly correlated. **(A)** Western blot was performed on total brain homogenates before extraction and insoluble fractions after extraction for the remaining three batches which include 9 control, 9 AD, 5 AsymAD, and 9 PD cases. TDP-43 was blotted as loading control. **(B)** Scatter plot of insoluble pT231-tau western blot band intensities across all four groups. The AD group is the only group significantly changed from control (*p*-Value ≤ 0.0001, Dunnet’s multiple comparisons test). **(C)** Group-wise scatter plot of insoluble U1-70K western blot. Both AsymAD (*p*-Value = 0.0427) and AD (*p*-Value = 0.0010) are significantly changed from control (Dunnet’s multiple comparisons test).

Supplementary Figure 2 Comparison of missing values in PRM targeted method and label free quantitation (discovery mode) untargeted method data. **(A)** A Venn diagram shows 324 common proteins in both PRM and data dependent acquisition (label free quantitation) method data. **(B)** Histogram of the percent of proteins with different levels of percent missing measurements for the 324 common proteins in PRM (*blue bars*) and untargeted Consensus LFQ (*yellow bars*) modes of analysis. Approximately 23% of the shared 324 proteins were removed in the untargeted LFQ analysis, while zero were removed in this current PRM analysis.

Supplementary Figure 3 Parallel reaction monitoring analysis and peptide peak quantification confirms known changes of insoluble tau in AD at the peptide level. **(A)** MS/MS spectra of representative IGSTENLK peptide of tau. The *x*-axis is the m/z of each fragment ion and the *y*-axis displays the relative abundance of each fragment ion. The top-5 product ions were labeled and highlighted in different colors. The spectra was obtained from AD sample E06-155. **(B)** Corresponding chromatography of top-5 product ions of IGSTENLK peptide across GPS, control, AsymAD, AD and PD cases where *x*-axis is retention time and *y*-axis is intensity. Each ion was represented by a different color. **(C)** Scatter plot of western blot band intensities and PRM quantification of VAVVRT[+80]PPK (AA226-234) pT231-tau peptide in CTL (*magenta*), AsymAD (*turquoise*), AD (*green*), and PD (*orange*) samples. Significance of correlation is calculated between the two independent measurements of pT231-tau (Student’s p for Pearson rho). **(D)** Scatter plot of western blot band intensities and PRM quantification of U1-70K protein. Significance of correlation is calculated between the two independent measurements of U1-70K (Student’s p for Pearson rho).

Supplementary Figure 4 The distribution of standard peptide response (standard peptide 2-5, isotope 3) across 50 runs before and after normalization. **(A)** The standard peptide intensity from each sample was averaged to 1 and plotted across all 50 runs. The *x*-axis indicates run number and *y*-axis displays the response. Theoretically, each of all 50 runs should exhibit the same response since an identical amount of standard peptides was added to each sample. However, due to systematic signal depreciation, the response gradually decreased before normalization. **(B)** After normalization, influence of technical variation was statistically eliminated with a final coefficient of variance (CV) of 14.69% by applying a correcting factor to each datapoint.

Supplementary Figure 5 Bicor coefficients rank RBPs according to correlation with Aβ and tau. **(A)** The 385 RBPs were ranked by their correlation to Aβ_(1–42)_ insolubility across all 44 cases examined. Each point is colored according to module membership of that RBP. The top 10 positively- and anti-correlated proteins are listed with their bicor coefficient value. **(B)** 385 RBPs were ranked by their correlation to tau insolubility across all 44 cases examined. Each point represents an individual RBP colored according to module memberships. The top 10 positively- and anti-correlated proteins are listed with their bicor coefficient value.

Supplementary Table 1 Summary of case information.

Supplementary Table 2 Sarkosyl-soluble and -insoluble protein yields.

Supplementary Table 3 Protein bands quantification of insoluble tau and U1-70K.

Supplementary Table 4 Correlation of pT231-tau/U1-70K quantification between western blot and PRM.

Supplementary Table 5 Raw PRM quantified peptide peak area before normalization.

Supplementary Table 6 Normalized peptide peak areas.

Supplementary Table 7 Peptide differential abundance analysis (imputed missing values marked in blue, log_2_-transformed, *t*-test).

Supplementary Table 8 Peptide differential abundance analysis (ANOVA).

Supplementary Table 9 Normalized protein quantification.

Supplementary Table 10 Normalized protein quantification (log_2_-transformed and regressed for age, sex, and PMI).

Supplementary Table 11 Protein differential abundance analysis (*t*-test).

Supplementary Table 12 Protein differential abundance analysis (ANOVA).

Supplementary Table 13 Z-score table for module clustering by GO terms.

Supplementary Table 14 Module assignment information.

Supplementary Table 15 Correlation of all insoluble RNA-binding proteins quantified to Aβ_(1–42)_ and tau.
