# Supplementary figures and images for: Targeted Quantification of Detergent-Insoluble RNA-Binding Proteins in Human Brain Reveals Stage and Disease Specific Co-aggregation in Alzheimer’s Disease
Source: Front Mol Neurosci. 2021 Mar 18;14:623659. doi: 10.3389/fnmol.2021.623659 (PMC8014091; doi:10.3389/fnmol.2021.623659)

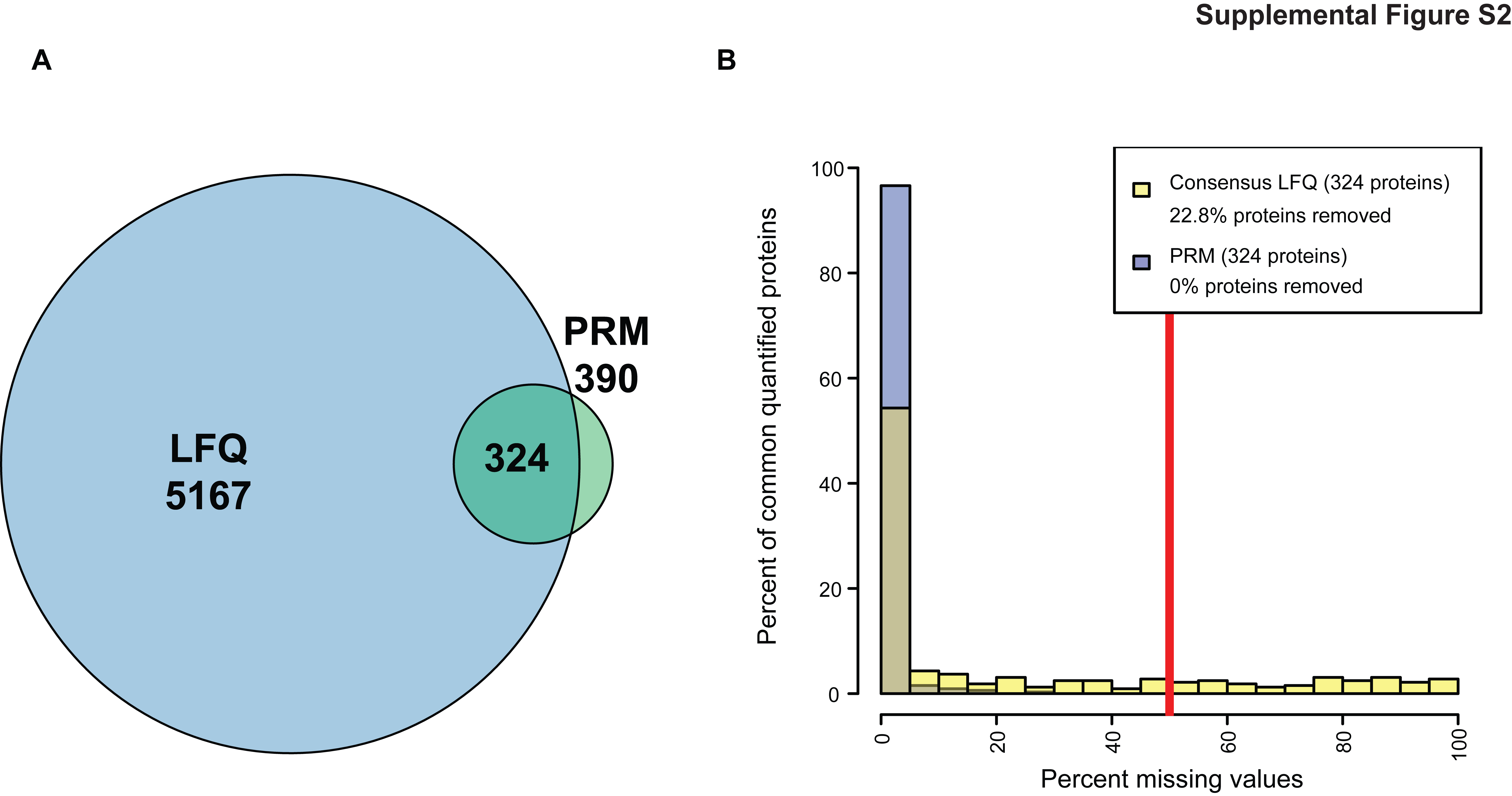

Supplement: Supplementary Figure 2 — Comparison of missing values in PRM targeted method and label free quantitation (discovery mode) untargeted method data. (A) A Venn diagram shows 324 common proteins in both PRM and data dependent acquisition (label free quantitation) method data. (B) Histogram of the percent of proteins with different levels of percent missing measurements for the 324 common proteins in PRM (blue bars) and untargeted Consensus LFQ (yellow bars) modes of analysis. Approximately 23% of the shared 324 proteins were removed in the untargeted LFQ analysis, while zero were removed in this current PRM analysis. [file Image_2.TIF]

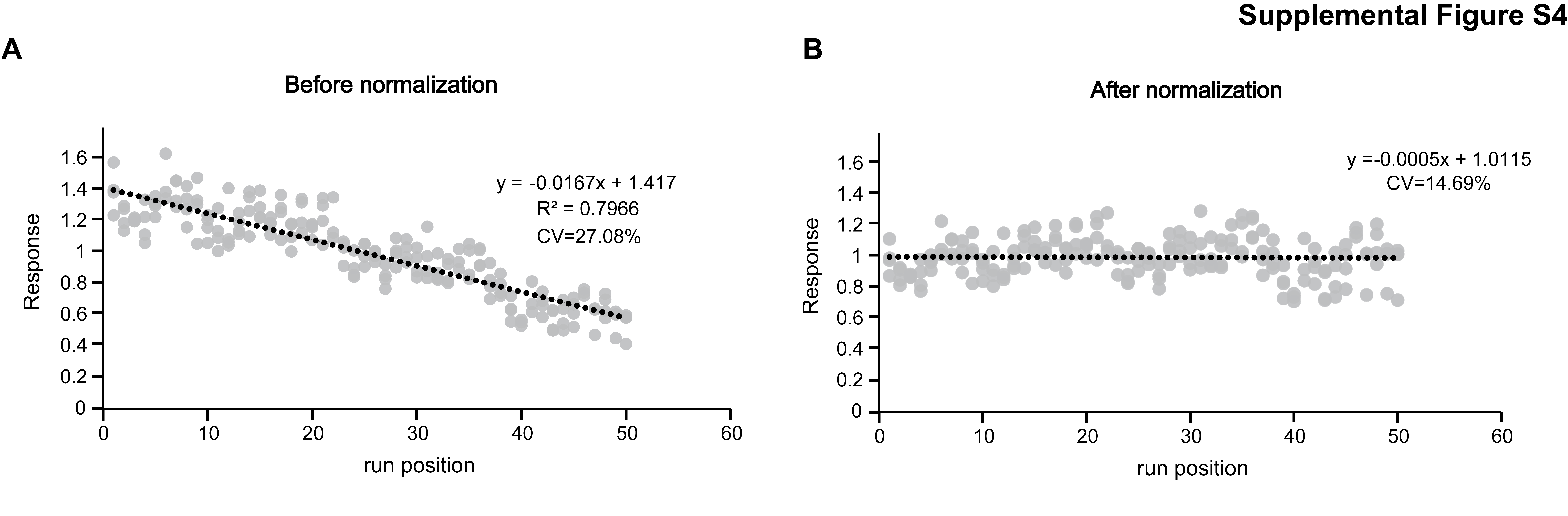

Supplement: Supplementary Figure 4 — The distribution of standard peptide response (standard peptide 2-5, isotope 3) across 50 runs before and after normalization. (A) The standard peptide intensity from each sample was averaged to 1 and plotted across all 50 runs. The x-axis indicates run number and y-axis displays the response. Theoretically, each of all 50 runs should exhibit the same response since an identical amount of standard peptides was added to each sample. However, due to systematic signal depreciation, the response gradually decreased before normalization. (B) After normalization, influence of technical variation was statistically eliminated with a final coefficient of variance (CV) of 14.69% by applying a correcting factor to each datapoint. [file Image_4.TIF]

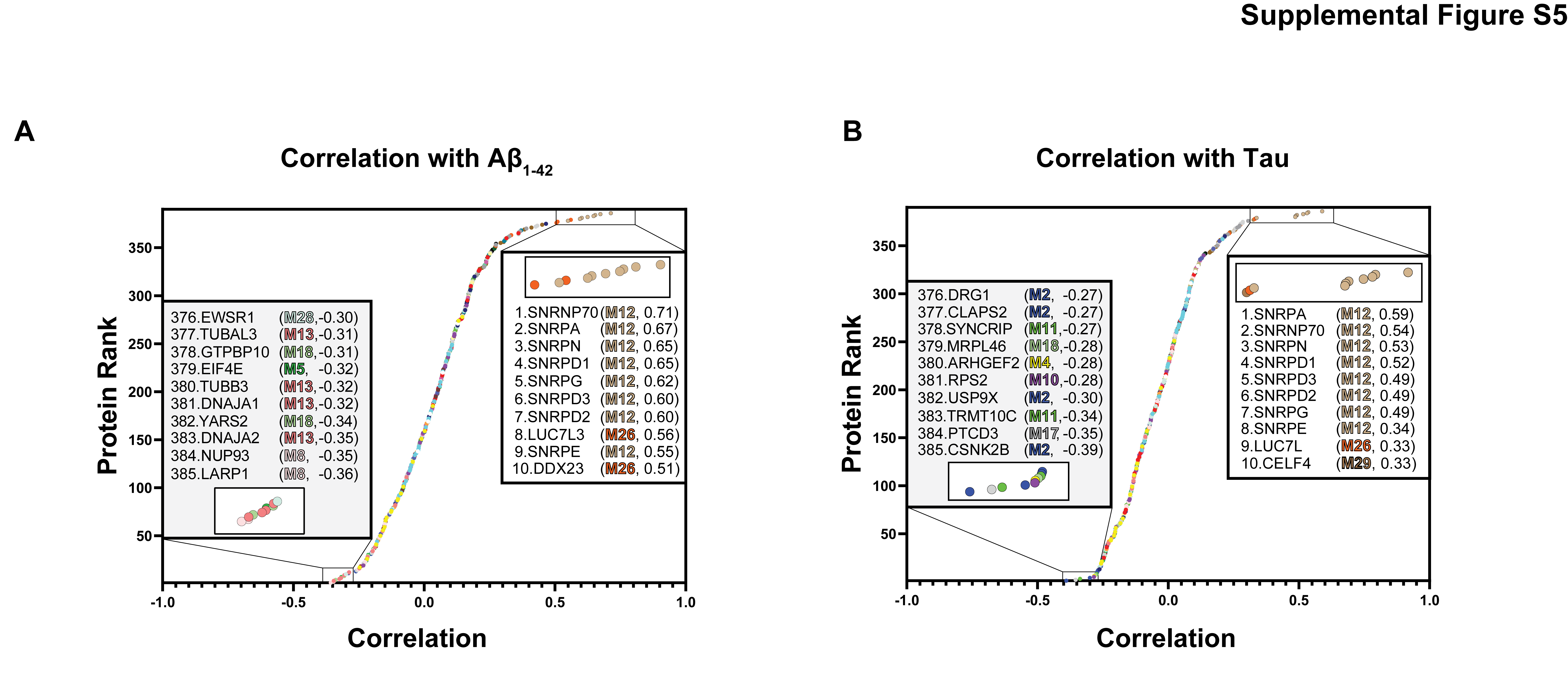

Supplement: Supplementary Figure 5 — Bicor coefficients rank RBPs according to correlation with Aβ and tau. (A) The 385 RBPs were ranked by their correlation to Aβ(1–42) insolubility across all 44 cases examined. Each point is colored according to module membership of that RBP. The top 10 positively- and anti-correlated proteins are listed with their bicor coefficient value. (B) 385 RBPs were ranked by their correlation to tau insolubility across all 44 cases examined. Each point represents an individual RBP colored according to module memberships. The top 10 positively- and anti-correlated proteins are listed with their bicor coefficient value. [file Image_5.TIF]
